# Gastrocnemius muscle herniation as a rare differential diagnosis of ankle sprain: case report and review of the literature

**DOI:** 10.1186/1754-9493-6-5

**Published:** 2012-03-14

**Authors:** Greta Bergmann, Bernhard D Ciritsis, Guido A Wanner, Hans-Peter Simmen, Clément ML Werner, Georg Osterhoff

**Affiliations:** 1 Department of Surgery, Division of Trauma Surgery, University Hospital Zürich, Zürich, Switzerland

**Keywords:** Gastrocnemius muscle herniation, Mesh graft repair

## Abstract

**Background:**

Muscle herniation of the leg is a rare clinical entity. Yet, knowing this condition is necessary to avoid misdiagnosis and delayed treatment. In the extremities, muscle herniation most commonly occurs as a result of an acquired fascial defect, often due to trauma. Different treatment options for symptomatic extremity muscle herniation in the extremities, including conservative treatment, fasciotomy and mesh repair have been described.

**Case presentation:**

We present the case of a patient who presented with prolonged symptoms after an ankle sprain. The clinical picture showed a fascial insufficiency with muscle bulging under tension. Ultrasound and MRI imaging confirmed the diagnosis of muscle hernia of the medial gastrocnemius on the right leg. Conservative treatment did not lead to success. Therefore, the fascial defect was treated surgically by repairing the muscle herniation using a synthetic vicryl propylene patch.

**Conclusions:**

Muscle hernias should be taken into consideration as a rare differential diagnosis whenever patients present with persisting pain or soft tissue swelling after ankle sprain. Diagnosis is mainly based on clinical aspect and physical examination, but can be confirmed by radiologic imaging techniques, including (dynamic) ultrasound and MRI. If conservative treatment fails, we recommend the closure with mesh patches for large fascial defects.

## Background

Muscle herniation in the extremities is a rare clinical entity. Most commonly, it occurs as a result of an acquired fascial defect, i.e. after trauma [1]. In symptomatic patients, there can appear pain or discomfort on physical exertion of the affected limb, but also paresthesia or the like by compression of nerves. It is, however, important to note, that the true incidence of the condition of muscle herniation of the lower extremities remains unclear. Many of these herniations are asymptomatic or may be misdiagnosed, e.g. a soft tissue tumor or successfully treated as another condition[2,3]. Often, even MRI findings are non-specific detecting subtle fascial and muscle signal changes[4].

Different treatment options for symptomatic extremity muscle herniation in the lower limb have been described [5-9]. These techniques were mostly used for tibialis anterior muscle herniation and include conservative management (activity limitation, compressive stockings...) as well as fasciotomy, direct approximation of the fascial defect, tibial periosteal flap, partial muscular excision, and patch repair with autologous fascia lata, [9]or synthetic mesh[6,7].

Up to now, however, the correction of symptomatic muscular hernia in the lower extremity using a synthetic mesh has been reported only in few case reports[6]. Only one case of gastrocnemius muscle herniation was published by Hong et al. in 2007[10].

We present a case of a patient who presented with prolonged symptoms after ankle sprain. He was diagnosed with fascial hernia of the medial gastrocnemius on the right leg. The muscle herniation was treated surgically by repairing the fascial defect using a synthetic vicryl propylene composite patch (Vipro II^®^, Ethicon, Neuchatel, Switzerland).

## Case presentation

A 42 year old patient presented to our hospital two months after he had distorted the right lower leg while climbing up the stairs. Initially, he had noticed a torrential sound and suffered from immediate pain with immobilisation. He presented with persisting pain, especially during exercise. The conservative treatment with activity limitation, partial weight bearing and compression stockings were not of success. When he was referred to our outpatient clinic half a year after trauma, the patient reported persisting spasmodic pain and swelling of the medial right lower leg. Only 300-400 m of walking were possible.

Clinical investigation showed a distinct pressure pain in the area of the right medial calf as well as muscle stiffness, swelling and a tender muscle bulge of the medial gastrocnemius muscle, which increased on physical examination i.e. in tension of the M. triceps surae. Bulging through the fascia appeared at the right medial gastrocnemius when tiptoeing. When relaxed, a soft spot was palpable at the same area. Additionally, the patient reported a hyposensitivity and hypalgesia of the right leg which was not dermatome related. Reflexes and motor function were normal, no Tinel-phenomenon could be found. Peripheral blood circulation was normal.

The measurement of lower leg circumference revealed 46 cm for the right leg and 45 cm for the left leg. Exercise did not influence the circumference; however the measurement of intracompartmental pressure in the superficial posterior compartment of the thigh showed an increase on the affected size from 40 cm H2O to 80 cm H20 while remaining 40 cm H2O on the healthy side.

The ultrasound showed subcutaneous adipose tissue imbibitions with fluid lamellae at the right medial lower leg. There were no signs for an abscess, seroma or hematoma.

**Figure 1:**
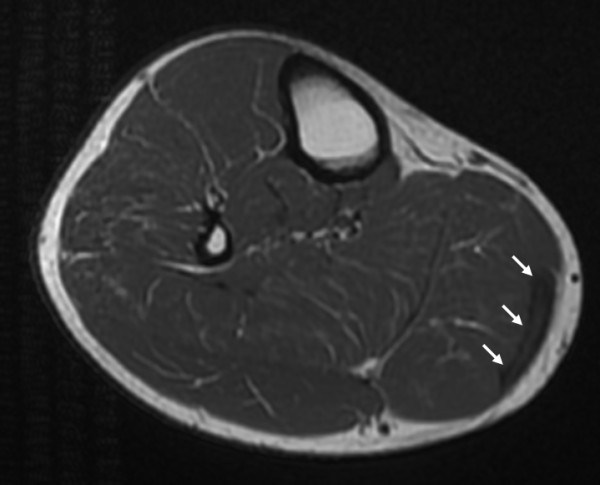
**MRI of the right lower leg**. Arrows pointing at a superficial hypointense lesion of proximal third of the medial gastrocnemic muscle.

Further neurologic electrophysiological investigation revealed no lesion of the sciatic nerve, polyradicular syndrome or spine canal stenosis. An MRI showed a superficial hypointense lesion of the medial gastrocnemius muscle in the proximal third, which was interpreted as a superficial scar of the muscle, possibly the status post muscle rupture by the radiologist (Figure 1). Yet, as the clinical picture showed a fascial insufficiency with muscle bulging under tension, it finally was explained by a fascial defect.

### Operative technique and follow up

**Figure 2:**
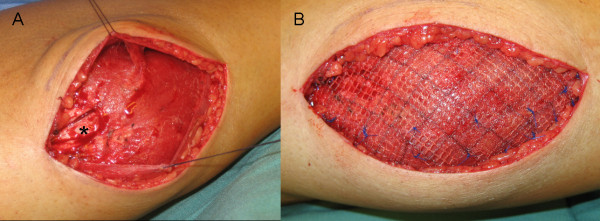
**Intraoperative photographs**. (A) Picture of the right calf after opening of the compartment. Note the scar tissue (*) adherent to the medial belly of the gastrocnemic muscle. (B) After partially reducing the fascial defect a composite Vicryl-Prolene mesh was inserted in inlay-technique.

The operation was performed under general anaesthesia with the patient supine. First, the muscle herniation was palpated at the right medial gastrocnemius. A longitudinal skin incision was made directly above the induration and the fascia was exposed. A thinning of the fascia was seen on an area of 3 × 7 cm. The compartment was opened (Figure 2) and partial resection of the scar tissue was performed for histological investigation. Intraoperatively, no signs of compartment syndrome (i.e. bulging after fascial opening) were seen.

The operative repair was achieved using a composite Vicryl-Prolene mesh (Vipro 2, Ethicon, Neuchatel, Switzerland) as an inlay. The mesh was secured in place under minimal tension with single button stitches (Prolene 3-0, Ethicon, Neuchatel, Switzerland).

The fascial defect could be reduced to 50% of the original size. Functionality of the muscle could be obtained. Skin closure then was performed in two layers.

Postoperatively, there were no complications, such as wound/mesh infection or thrombosis. No signs of a compartment syndrome were seen. Histological examination of the intraoperative biopsy revealed collagen connective tissue with low inflammation and perivascular fibrosis consistent with muscular scar tissue.

The follow up treatment included a short leg cast, restriction of physical loading and prevention of thrombosis with low molecular weight Heparin. Two weeks postoperatively, the patient still had pain in the right lower leg. Additionally, he complained about tenderness around the insertion of the tendon of the tibialis anterior muscle. After Xylocain injection, these symptoms were interpreted as tendonitis of the tibial anterior muscle caused by cast. There was also pressure pain in the scar region. Therefore, the analgetic and anti-inflammatory therapy, physiotherapeutic treatment with lymphatic drainage and mobilization of the lower extremity with full physical loading were continued.

**Figure 3:**
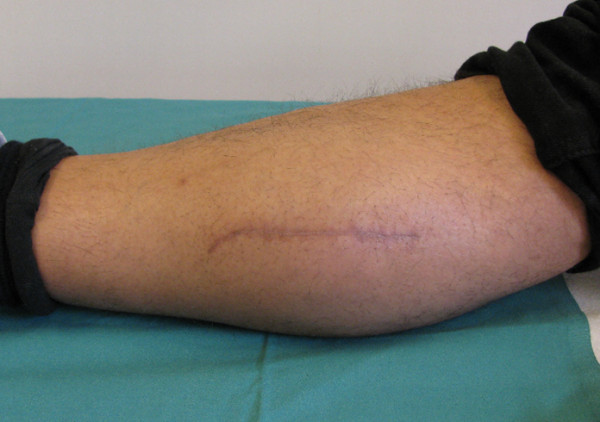
**Follow up three months postoperatively**. Scar over the right medial gastrocnemicus without bulging or signs of inflammation.

At 6 weeks, the pain both in the region of the tibialis anterior muscle tendon and in the scar region had markedly decreased. The walking distance had increased to approximately 300 m and the patient was fully employable. The pain was remarkably less than preoperatively. Six weeks postoperatively, an MRI showed the scar with perifocal fluid with no proof of residual muscle herniation. Two (Figure 3) and three months postoperatively complaints further decreased.

Six months after surgery, the patient was admitted to our out patient clinic by his general practitioner because of recurrent pain in the same leg. In the examination this pain appeared rather to be of radiating character, however. An MRI of the lumbar spine showed osteochondrosis of L3/L4 and, subsequently, an infiltration of the L3 root brought pain relief.

## Discussion

Muscle hernia can be defined as a focal protrusion through a fascial defect[1]. This entity was first described by Ihde in 1929[1]. Since then, there have been various reports on this issue [5-8,11-15]. Therefore, herniation of muscles are not uncommon in the leg and with increasing physical activeness, orthopaedists will encounter more of such cases[16]. Yet, because of lacking recent research, the actual incidence of symptomatic muscle herniation remains unknown.

Many of the cases report muscle herniation of the leg, especially the thigh. Hereby, the tibialis anterior hernia is most common and has been described in various cases [5,8,9,13,14,17-20]. Muscular hernias of the posterior compartment, however, are rare. There is so far only one case reported involving the gastrocnemius muscle[21]. Usually, muscle hernias are not symptomatic, yet, they may present with cramping or pain[5,14,17,22]. Our patient reported pain with sensory disturbance at the lower leg.

Therefore, our case of a herniation of the medial gastrocnemius head is unusual.

Muscle hernias can be divided into two groups: constitutional and traumatic. Traumatic hernias occur either after direct or indirect trauma. In direct traumata, the fascia itself is injured resulting e.g. from fractures, wounds or contusion. Furthermore, hernias can appear secondary to increased intracompartmental pressure. Indirect trauma means injury to the contracted muscle that can cause rupture of the fascia[23]. In our case the patient's history correlated well with an indirect traumatic muscle herniation and compartment pressure was pathologic only under exercise. As preoperative intracompartemental pressure measurements were normal in rest, an exercise induced compartment syndrome due to space limitation by the scar tissue was hypothesized. Thus, we decided for surgical scar tissue resection and - in the same intervention - tension free mesh covering to avoid painful recurrence of the muscle hernia.

Patients with muscle hernia usually suffer from pain or present due to cosmetic reasons or concerns of having a tumor[5]. In our case, the patient reported persisting pain as well as hyposensitivity on the thigh and lower leg. These complaints can not be fully explained by the hernia of the gastrocnemius muscle. Alhadeff et al. reported an unusual case of pseudoradicular symptoms caused by compression of the common peroneal nerve in the popliteal area by gastrocnemius muscle herniation. The patient suffered from pseudoradicular symptoms that resembled sciatica[21]. Therefore, neurologic symptoms resembling the symptoms of our patient are possible in patients with hernia occurring at the site of nerve perforation of the fascia[16].

Radiologic imaging techniques, including MRI and CT and ultrasound have been used to have the definitive diagnosis of muscle herniation and to identify the defect[5,10,24,25].

Dynamic sonography must be the first imaging examination due to its low cost and ready availability [11]. However, as seen in our case, a fascial thinning is sometimes difficult to detect. It has been suggested the use of dynamic MR imaging in the evaluation of suspected muscle herniations to better delineate the fascial defect and the size of the muscle herniation, if dynamic sonography does not adequately define these features [26,27]. It was hypothesized that MRI can be useful in planning operative treatment [28]. We used MRI to localize and confirm pathology in the area of pain.

Treatment of muscular hernias is mainly dependent on clinical symptoms and reaches from conservative measures to operative intervention. Asymptomatic hernias usually require no treatment or can be treated conservatively[16]. For mild cases, a support stocking, can be of benefit along with rest and activity modification [1]. For patients with stronger symptoms or those in whom conservative treatment has failed to improve symptoms, operative methods can be considered[5].

In our case, conservative methods were not able to alleviate the symptoms of the patient and therefore operative treatment was indicated.

There are different operative procedures, including direct repair [29,30] fascial grafting, [9,23,31,32] fasciotomy [5,16,18]and more recently, mesh grafting[6,7].

Direct repair is possible when the defect is small and the laxity of the borders permits approximation; this has been practiced in the past [23]. However, because of reports of compartment syndrome after direct repair, [5,8] this method should only be used when the defect is small and essential close postoperative follow up is assured [16]. Some authors consider the longitudinal fasciotomy the safest method of treatment [5,11,16,18,29,33]. In our case, the fascial defect was 3 × 7 cm and therefore too large for direct repair; the compartment pressure was already increased preoperatively and a direct repair would have had a high risk of postoperative compartment.

There are several reports about successful results after fascial defect coverage using artificial meshes [6,7]. They suggest that the operative procedure is simple, more rapid, and less complicated than other techniques and can be used for large defects. The mesh is fixed above and not under the fascia allowing the underlying muscle to slide without any friction by the mesh or sutures[6,7].

Lee et al. reported a patient who had multiple herniation of the tibialis anterior muscle. Large defects of the fascia were repaired using a mesh [17]. In our case, we performed a mesh grafting repair of the fascial defect with reduction of the fascial defect by 50%. A compartment syndrome as the typical complication after repair of fascial defects was not seen[8,18].

We used a Vicryl-Propylene mesh. Using a permanent mesh in the treatment of hernia is advantageous because it is robust and very durable. Vicryl-Propylene composite meshes are made from 50% resorbable vicryl and 50% non-absorbable Polypropylene. It is therefore partially resorbable and makes a tension free fixation possible. Potential disadvantages may be an increased risk of infection as there is a synthetic nonabsorbable foreign body and there is the risk of adhesion between the mesh and the underlying structures.

The outcome in the follow up examination after 6 weeks showed a decrease of the preoperative pain and a good functionality with full physical load, complete mobilization and 100% employability postoperative. It can be assumed that approximately 2 months are needed to induce a stabile scarring. This is similar to the results of Siliprandi et al. [6], who could show that this procedure can provide good functional results and a good cosmetic appearance without complications and sequelae. Results from various studies point towards a good long time outcome without recurrence of the hernia also after years[7].

Finally, a muscle hernia has not always to be painful and other reasons causing the complaints have to be excluded before surgery. In our case, the patient developed a tendinitis of the tibialis anterior muscle after two weeks and a radiculopathy of L3 that were both probably caused by the abnormal bearing due to lasting restriction of physical loading and immobilization.

## Conclusions

We report the case of a 3 × 7 cm symptomatic fascial hernia after ankle sprain that was successfully surgically repaired by using a Vicryl-Propylene mesh. Potential muscle hernias have to be taken into differential diagnostic considerations in patients with persisting pain or swelling after distortions of the ankle joint. If a patient is not symptom-free under conservative treatment, surgical repair with mesh graft can be performed with relatively low-risk.

## Competing interests

The authors declare that they have no competing interests.

## Authors' contributions

GB participated in the clinical examinations and drafted the manuscript. BDC and CMLW were involved in the clinical examinations and the surgical procedures and revised the manuscript. GAW and HPS revised the manuscript. GO participated in drafting the manuscript. All authors read and approved the final manuscript.

## Consent

Written informed consent was obtained from the patient for publication of this Case report and any accompanying images. A copy of the written consent is available for review by the Editor-in-Chief of this journal.
